# A Digital Atlas of Middle to Large Brain Vessels and Their Relation to Cortical and Subcortical Structures

**DOI:** 10.3389/fnana.2016.00012

**Published:** 2016-02-17

**Authors:** Roberto Viviani

**Affiliations:** ^1^Institute of Psychology, University of InnsbruckInnsbruck, Austria; ^2^Psychiatry and Psychotherapy Clinic III, University of UlmUlm, Germany

**Keywords:** digital atlas, brain vasculature, brain anatomy, time-of-flight angiography, brain perfusion

## Abstract

While widely distributed, the vascular supply of the brain is particularly prominent in certain anatomical structures because of the high vessel density or their large size. A digital atlas of middle to large vessels in Montreal Neurological Institute (MNI) coordinates is here presented, obtained from a sample of *N* = 38 healthy participants scanned with the time-of-flight (TOF) magnetic resonance technique, and normalized with procedures analogous to those commonly used in functional neuroimaging studies. Spatial colocalization of brain parenchyma and vessels is shown to affect specific structures such as the anteromedial face of the temporal lobe, the cortex surrounding the Sylvian fissure (Sy), the anterior cingular cortex, and the ventral striatum. The vascular frequency maps presented here provide objective information about the vascularization of the brain, and may assist in the interpretation of data in studies where vessels are a potential confound.

## Introduction

Digital brain atlases are an increasingly important instrument in neuroimaging research, as they provide a spatial framework for the selection, interpretation, and generalization of complex experimental data (Mazziotta et al., 2001; Toga et al., 2006; Van Essen and Dierker, 2007). Here, we present a digital atlas of middle to large vascular structures based on MRI data acquired with the time-of-flight technique (TOF) on 38 healthy individuals. TOF magnetic resonance angiography uses a short echo time and differences in saturation of inflowing and stationary spins to make blood much brighter than tissue without resorting to an invasive contrast medium (Nishimura, 1990; Wehrli, 1990). Therefore, in TOF images vessels are staked out by higher signal intensity against a gray background.

The purpose of the present work is to provide objective information to experimenters about the location of large vessels. Volume-based atlases in common use present a picture of a uniform, almost gapless partition of the brain volume into areas or anatomical structures. In some studies, however, interpretation of findings may benefit from knowledge of where vessels are likely to be present. In functional MRI (fMRI) studies, heart pulsation is known to affect the signal near large arterial vessels (Dagli et al., 1999; Lund et al., 2006), and the tendency of the BOLD signal to be displaced towards large venous sinuses complicates the localization of task effects (Lai et al., 1993; Menon et al., 1993). In the study of cortical connectivity (Margulies et al., 2007; Biswal et al., 2010), identifying the confounding signal arising from the interplay of heart beat and acquisitions is an essential methodological task (Cordes et al., 2001; Margulies et al., 2010; Van Dijk et al., 2010). In perfusion imaging, the vascular tree constitutes a well-known source of signal covariance (Viviani et al., 2009, 2010) due to factors such as transit time effects (Alsop and Detre, 1996) or the direct effect of blood flow on the signal (Dagli et al., 1999). More generally, region of interest (ROI) selection (Poldrack, 2007) or the precise measurement of the signal in crucial locations associated with disease or predictive of treatment outcome (Mayberg et al., 1997; Kirchheiner et al., 2010) may require careful assessment of the possible role of vascular confounds.

## Materials and Methods

### Data Acquisition

**Figure 1:**
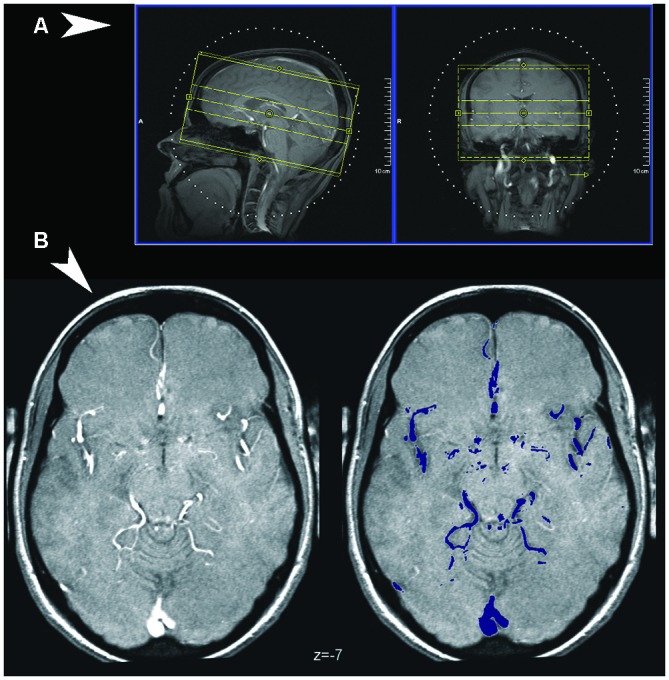
**(A)** Coverage of the Time-of-Flight (TOF) sequence, visualized over the scout localizer at the console of the Siemens scanner used in the study (Syngo software). **(B)** Detection of vessels in TOF images. An illustrative normalized TOF image (left), arbitrarily chosen as the first from the series, and the major vessels as detected by thresholding the volume (in color on the right).

The study was approved by the Ethical Committee of the University of Ulm, Germany, as part of a larger database project for the collection of structural images (Viviani et al., 2007, 2009). After giving written informed consent, healthy participants were scanned with a 3T Siemens Allegra scanner at the Department of Psychiatry and Psychotherapy III of the University of Ulm. TOF angiography images were obtained from 42 healthy volunteers. Data required for coregistration were lost for one participant, and further three participants were excluded due to movement artifacts. The final sample included 38 participants aged between 18 and 59 (mean age 29.1, standard deviation 9.6, 14 males). Acquisition of TOF data is constrained in the number of slices, due to the amount of transferred energy and the relatively long acquisition times. The sequence used in the present study differs from a standard clinical TOF in that no saturation band was present in the upper part of the cerebrum. This band intentionally suppresses signal from venous sinuses, which is present in our images. Although perhaps an undesirable aspect of the present atlas, only by removing the suppression band was it possible to obtain images from the upper half of the cerebrum. There was no evidence that other large venous collectors situated further downstream, such as the jugular veins, provided any signal in our TOF images (more time was available for water proton spins to reach equilibration). The number of slices was increased to obtain a larger coverage of brain volume than in typical clinical applications (Figures 1A,B). Images were acquired in a transverse oblique orientation, parallel to the subcallosal line (Figure 1A), which is the line that joins the undersurface of the rostrum (front) and splenium (back) of the corpus callosum (Simon et al., 2006). To limit SAR exposure, the scan was carried out in five separate sections (blocks) linked together by the scanner software. Details of the acquisition protocol are given in the Table 1. For coregistration, sagittal T1-weighted high-resolution MPRAGE volumes were acquired (image size 256 × 256 × 208 voxels, voxel size 1 × 1 × 1 mm, TR/TE 3300/96, flip angle 90°, bandwidth 1220 Hz/pixel, field of view 240 × 240; see Viviani et al., 2007).

**Table 1:** **Image acquisition parameters**.

Parameter	Value
TR	41 ms
TE	4.92 ms
Orientation	Transversal
Phase encoding	Right-left
Phase oversampling	0%
3-D blocks	4
Slices in block	16
Slice oversampling	25%
Distance factor	−37.50%
Field of view	190 mm/80%
Slice size	1.8 mm
Flip angle	25°
Bandwidth	112 Hz/Pixel
Voxel size	0.3 × 0.3 × 18
Image size	512 × 640 × 56
Acquisition time	7 min 44 s

### Data Processing

Average slice signal intensity of TOF images was preliminarily set to the volume mean after skull stripping (Dogdas et al., 2005) with the package BrainSuite version 9.01 (Shattuck and Leahy, 2002)^1^, one of the software tools distributed by the International Consortium for Brain Mapping (ICBM)^2^. The same software was used to apply a diffusion filter (six iterations, diffusion constant 160, edge detection constant 0.62; for rationale, see Perona and Malik, 1990) and local intensity correction (default parameters, histogram radius 12, sample spacing 16, control point spacing 60, spline stiffness 0.001). To normalize the data (Friston et al., 1996), we applied to TOF images the parameters of a volume-based normalization of concurrently acquired high-resolution T1-weighted data computed with the software package SPM5^3^. After a 12-parameter affine registration, the normalization algorithm employed nonlinear warps described by a linear combination of low spatial frequency discrete cosine basis functions with cutoff 25 mm to minimize the discrepancy with the Montreal Neurological Institute (MNI) template brain as quantified by a square difference objective function (Ashburner and Friston, 1999). Preliminary coregistration of TOF to T1-weighted images was obtained with the same package. This procedure is analogous to a common strategy for the normalization of echo-planar imaging data. Images were resampled to a voxel size of 0.3 × 0.3 × 1 mm for high-definition images used in the figures. For convenience, the digital atlas is also provided at resolution of isotropic voxel sizes of 1 and 2 mm.

To generate vascular frequency maps indicative of the presence of a partial volume effect, the normalized images were thresholded at the 97th percentile of the TOF signal. This threshold was selected empirically as the one that could select most vessels in the TOF images without selecting nonvascular tissue (see Figure 1B). Suprathreshold signals of nonvascular origin such as those from the orbital area and skull were corrected in each individual volume using a semi-automatic procedure, based on visually identifying clusters of confounding signal. Suprathreshold voxels were subsequently given the value 1, and averaged across volumes to provide the overlay data in the atlas, which express, in percent, the incidence of vasculature in the sample. Illustrations were prepared with the freely available software MriCroN by Chris Rorden^4^. Underlay images were obtained from the high-resolution ICBM atlas (Lancaster et al., 2000) of the McConnell Brain Imaging Center (asymmetric version, isotropic size 0.5 mm; Fonov et al., 2011).^5^ All coordinates are in MNI space (Evans et al., 1992).

## Results

Because of its high metabolic requirements, the brain is supplied by a dense arterial network (Kaplan and Ford, 1966). The focus here will be on the anatomical structures where vessels are particularly prominent either because of their size or their tendency to cluster.

**Figure 2:**
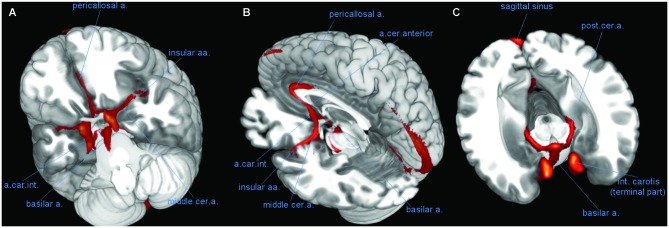
**Course of large vessels.** Three-dimensional rendering of the vascular frequency maps, thresholded at levels of 60% vascular frequency, showing the most likely course of the basilar artery (BA), the pericallosal artery (PC), and the insular arteries fanning out of the middle cerebral artery (CM). **(A)** View of the anterior face of brain from below, after removal of the most anterior part of the temporal and prefrontal lobes. **(B)** Latero-posterior view, after removal of most of the left hemisphere. **(C)** Likely course of the posterior cerebral artery (CP) along the medial border of the hippocampus (transversal slice, after removal of upper part of the brain). Abbreviations: a, artery; aa, arteries; int, internal; cer, cerebral; car, carotis.

The brain is supplied by the two internal carotid arteries and the basilar artery (BA), which results from the anastomosis of the two cerebral arteries. These vessels were localized consistently in TOF images, with voxels assigned to the vascular structure reaching frequency values of over 80% in correspondence to these vessels. Another important artery giving rise to high localization frequencies was the anterior cerebral artery and its prolongation, the pericallosal artery (PC) (Figure 2). MNI coordinates of peak localization frequencies for these and other large vessels are given in Table 2. For the internal carotid artery (CR), the peak frequencies (over 90%) were reached at coordinates −10, 5, −24 (96%) and 11, 3, −22 (95%). For the BA, the peak voxel was 0, −11, −23 (89%).

**Table 2:** **MNI coordinates of peak voxels assigned with high frequency to vessels**.

	*x*	*y*	*z*	Frequency (%)
Internal carotid	from ± 10 to ± 12	from 2 to 5	from −30 to −20	80–95
Basilar	0	−12	from −23 to −20	80–90
Anterior cerebral	0	12	−18	70
Anterior cerebral	0	25	3	85
Pericallosal	0	11	28	75
Middle cerebral L	−27	4	−15	75
Middle cerebral R	24	4	−15	65
Posterior cerebral	±15	−17	−16	70–75

*L, left; R, right; x, y, z: axes of MNI coordinates, in mm*.

The anterior cerebral and PC arteries are commonly present as a pair of left and right vessels, running side by side deep in the medial longitudinal fissure (Critchley, 1930). However, in many individuals there is just one artery for both hemispheres (azygos anterior cerebral artery) and more rarely up to four (Stefani et al., 2000). In the TOF data the peak frequencies for these arteries were reached on the midline.

### Amygdala, the Basal Forebrain, and the Striatum

Several anatomical structures in the ventral part of the brain are located in the neighborhood of large vessels (Lang, 2001). The anteromedial temporal lobe and the basal forebrain are bordered by the internal carotid, the middle, and posterior cerebral arteries. These vessels, in turn, give origin to smaller arteries that penetrate the brain parenchyma (anterior choroidal, central, and striatal arteries, Critchley, 1930; Feekes and Cassel, 2006). Both types of spatial relationship, neighborhood or interpenetration, may give rise to partial volume effects of vascular structures on brain parenchima or mixed signal in normalized images.

**Figure 3:**
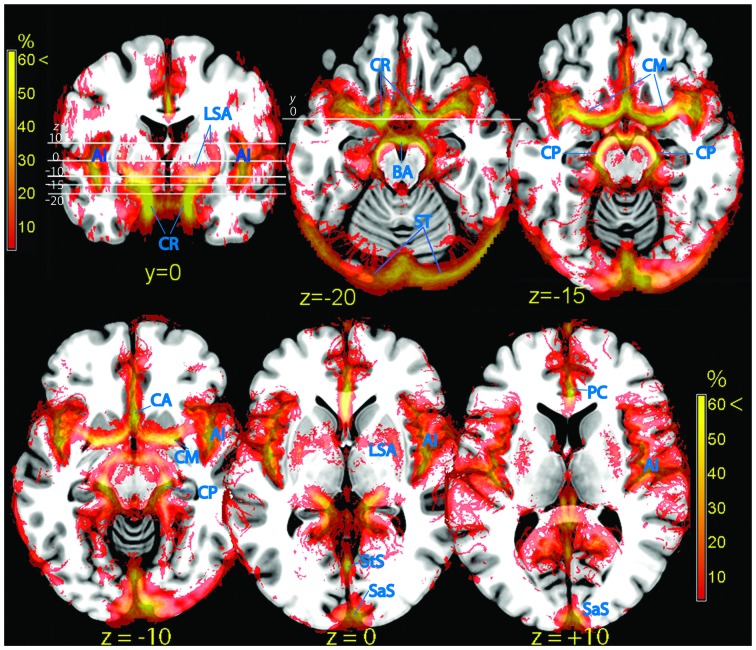
**Vascular frequency maps in an axial slice located just superior to the circle of Willis (top row), and at the level of the insula (bottom row), overlaid on the ICBM high-resolution T1-weighted template, showing the major outlets of the internal carotis (anterior, middle, and posterior cerebral arteries).** In the posterior part of the brain major sinuses are visible. The frontal section (top left) shows the internal carotis and the smaller vessels entering the ventral striatum from below. The high vascular frequency of the deep Silvian fissure, covering the insula, is also visible. Vessels (blue labels): LSA, lenticolo-striatal arteries; AI, insular arteries; CR, carotid artery; BA, basilar artery; ST, sinus transversus; CM, middle cerebral artery; CP, posterior cerebral artery; PC, pericallosal artery; StS, straight sinus; SaS, sagittalis sinus.

**Figure 4:**
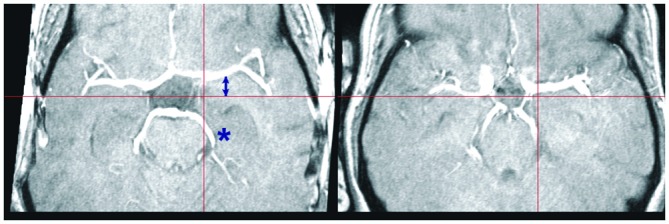
**Individual variation in large vessel course.** Transversal slices of two TOF images selected to illustrate individual variation in the course of the middle and posterior cerebral arteries. The crosshair selects the same MNI coordinates at *z* = −14. Note the displacement of the middle (arrow) and posterior cerebral arteries (asterisk) in these two subjects relative to the crosshair arms (red bars). Although the two brains are correctly normalized, as shown by the location of the notch in the brain profile corresponding to the Sylvian fissure, the position of the vascular tree depends on the precise shape of the brainstem and temporal lobes, which may differ considerably between individuals.

After overlaying the vascular frequency map obtained from TOF data on a transversal slice of the brain, parts of the amygdala and the parahippocampal cortex were shown to overlap with vascular structures such as the middle cerebral artery (CM; Figure 3, top row and *z* = −10 in the bottom row). This partial overlap may arise from individual differences in the size and shape of these limbic structures, which displace the vessels running close to their surface (Figure 4).

Signal arising from smaller penetrating vessels (central and striatal arteries) was particularly conspicuous in the ventral striatum (Figure 3, frontal section top left), the territory dorsal to the amygdala (ventral pallidum, substantia innominata). More dorsally, the vascularization of the pallidum was clearly visible (lenticulostriate arteries, Feekes and Cassel, 2006; Figure 3, bottom row).

### Hippocampus

**Figure 5:**
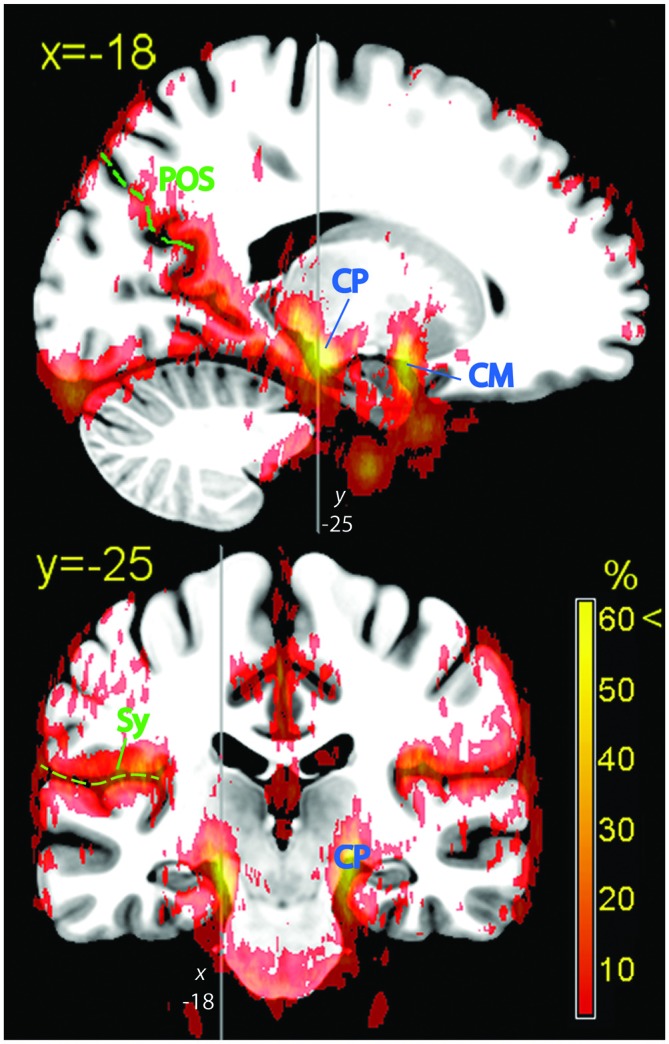
**Hippocampal formation.** Vascular frequency maps illustrating the course and hippocampal involvement of the posterior cerebral artery, overlaid on the high-resolution ICBM template brain. The disappearance of the sagittal sinus at the topmost part of the brain is due to artifactual loss of coverage. Vessels (blue labels): CP, posterior cerebral artery; CM, middle cerebral artery. In green: POS, parieto-occipital sulcus; Sy, Sylvian fissure.

The hippocampus is bordered by two large vessels: anteriorly, the internal CR and postero-medially, the posterior cerebral artery (CP; Duvernoy, 2005). These two large vascular structures partially overlapped with the medial part of the hippocampus at its anterior tip (Figure 3, top row) and at the height of the thalamus (Figure 3, bottom row, *z* = −10; Figure 5). In the majority of individuals, these regions are occupied by the entorhinal cortex and the subiculum, respectively (Amunts et al., 2005).

### Perisylvian Region

At the end of its course toward the sides of the brain, the CM bends backwards and gives origin to the insular arteries, a bundle of vessels running within the Sylvian fissure (Sy). The whole space separating the insular cortex from the frontal, parietal, and temporal operculi was characterized by fairly high vascular frequency (Figure 3, frontal section top left, and bottom row), especially in the ventral and posterior portions, corresponding to the inferior circular sulcus and the border between the posterior insula and the parietal operculum (Türe et al., 2000). The cortical areas most affected by the overlap were those of the posterior perisylvian region: the primary auditory cortex (Morosan et al., 2001), the parietal operculum (Eickhoff et al., 2006), and the posterior insula (Kurth et al., 2010). Anteriorly, the frontal operculum and Broca’s area/ventrolateral prefrontal cortex (BA 44, 45: Amunts et al., 1999), and to a less extent the ventral part of the primary sensory cortex colocalized with sparse vascular structures (Figure 3, bottom row).

### Medial Wall

**Figure 6:**
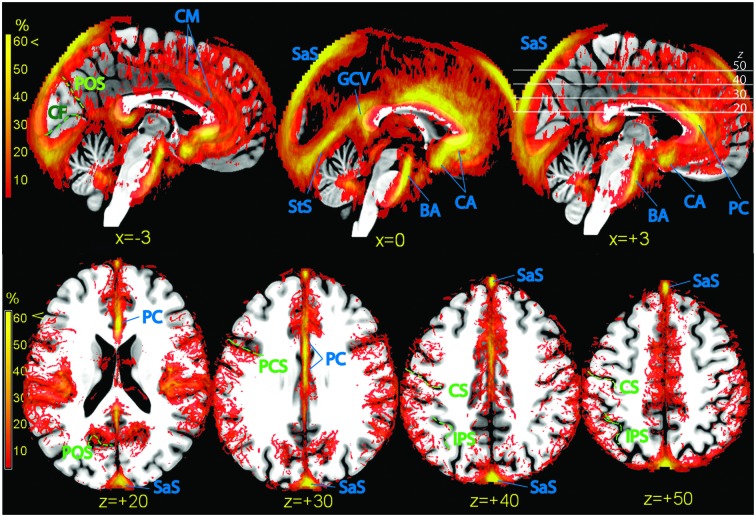
**Vascular frequency maps illustrating incidence of vasculature in the medial wall (top row) and in the superior portion of the brain (bottom row), overlaid on the high-resolution ICBM template brain.** The disappearance of the sagittal sinus at the topmost part of the brain is due to artifactual loss of coverage. Vessels and sinuses (blue labels): CM, region corresponding to the common course of the callosomarginal artery, when present; BA, basilar artery; CA, anterior cerebral artery; PC, pericallosal artery. SaS, sagittal sinus; StS, straight sinus; GCV, great cerebral vein. There is no consensus in the literature on point of transition between anterior cerebral and pericallosal artery. In green: POS, parieto-occipital sulcus; CF, calcarine fissure; PCS, precentral sulcus; CS, central sulcus; IPS, intraparietal sulcus.

The dense web of vascular structures running along the medial wall is displayed in Figure 6 (top row). Of particular note is the course of the anterior and pericallosal arteries encircling the genu of the corpus callosum (Critchley, 1930). When averaged across subjects, the course of these vessels displayed a tendency to deviate from the midline, so that the subgenual cortex colocalized more often with vascular structures on the left, and the supragenual cortex on the right. The incidence of vessels in the posterior cingular region was less conspicuous. In the region below and around the splenium the signal was sparse and symmetric (great cerebral vein, GCV). The cuneus and precuneus colocalized with signal arising from vascular structures running in the parieto-occipital sulcus (POS; precuneal arteries, Figures 5, 6).

Also the isocortex surrounding the anterior cingulus was bordered by a dense, poorly structured vascular web (Figure 6, top row). This region is characterized by high individual variability (Critchley, 1930; Ugur et al., 2006). While in some individuals (about 60%) there is an artery running dorsally and parallel to the pericallosal (callosomarginal artery), in others this artery is missing. Instead, arteries originate directly from the PC and take a radial course towards the periphery. In the maps of the top row of Figure 6, a tenuous intensification of vascular frequency is visible along the cingulate sulcus (Vogt et al., 1995), corresponding to a common path of the callosomarginal artery.

### Occipital and Calcarine Cortex

While the anteroventral part of the brain is characterized by the presence of large arteries, the main vascular structures in the posterior part of the brain are sinuses in the dura mater and tentorial tissue. The sagittal and transversal sinuses were by far the most prominent collectors of venous blood, followed by the straight sinus (StS, Figure 6, top row). The posterior arm of the sagittal sinus tended to localize either immediately left or right of the midline; in our sample, the tendency was to localize more often on the right. The sagittal and StS on the midline, and the transversal sinuses at the brain edge were partially colocalized with the occipital and calcarine cortex (Figure 6, bottom row).

### Superior Region

Axial slices of vascular frequency maps in the upper portion of the brain are shown in Figure 6, bottom row. A tendency is recognizable for vessels to be detected near the sulci of the ICBM template, notwithstanding the large individual variability of sulci. Also visible in Figure 6, bottom row are vessels running in the space between the cortex and the cranium, and whose distribution along the anteroposterior axis in uneven. Cortical pial vessels are of both arterial and venous nature, and the regional distribution observed here is consistent with description in the literature of the localization of pial vessels of largest caliber (Duvernoy et al., 1981).

### Description of the Atlas Data Available for Download as Supplementary Material

The atlas is made available in NIfTI format at the website^6^. Table 3 describes the volumes that may be downloaded. The main source of information is the atlas in the original high resolution. The volume contains the frequency of detection of vessels in our dataset, in percent, and may be used to visualize their course by overlaying it together with activation maps. Volumes are also provided after resampling at the isotropic resolutions of 1 and 2 mm, which are most commonly used in structural or functional studies. These volumes may be used to identify or create masks staking out regions where the vascular density is high.

**Table 3:** **Volumes made available as part of the atlas**.

Filename	Description	Resolution (mm)	Size (MB)
VesselDensityHR.nii.gz	High resolution vessel density atlas	0.3 × 0.3 × 1	3.0
VesselDensityMR.nii.gz	Medium resolution vessel density atlas	1 × 1 × 1	0.5
VesselDensityLR.nii.gz	Low resolution vessel density atlas	2 × 2 × 2	0.1
MeanHrTOF.nii.gz	Mean normalized TOF volume	0.3 × 0.3 × 1	25

*mm, millimetres; MB, megabytes*.

When using these data to interpret findings of other studies, it should be remembered that the quality of the data is limited by several factors. One is the low resolution in the vertical direction. This is a source of imprecision in the visualization of the relationships between vessels and brain tissues at oblique surfaces. A second limitation is the lack of resolution between arterial and venous vessels. A final issue is given by possible difficulties in redressing differences in brain shape induced by the geometry of the magnetic field of the original volumes, by the signal loss due to different susceptibility artifacts, and the possible use of customized normalization templates across specific studies and human populations. To enable users to assess and potentially address these discrepancies, the mean TOF normalized volume from all subjects in the study is provided in the atlas. This volume contains structural information that may be used to compute the cross-normalization to another template, or to renormalize the data using different registration parameters.

## Discussion

The involvement of anatomical structures with middle and large vessels displayed regional differences that were both quantitative (with some areas affected more than others) and qualitative. The quantitative differences staked out regions where the presence of large vessels was prominent. The qualitative differences were due to different relationships between vessel course and anatomical structures, which may be ordered along a range according to the extent of intermingling.

At one extreme of this range, large vessels were located in separate spaces well outside brain parenchyma, but individual variation in their course brought about a partial overlap between vessels and registered brain structures. This kind of overlap was exemplified by the relationship between the amygdala and the terminal part of the internal carotis and the CM. Even if among the least variable of all anatomical structures, the amygdala has a size that varies by a factor of two across healthy individuals (Amunts et al., 2005). The finding that a considerable portion of the superficial group of the amygdala overlapped with the course of the CM is consistent with differences in the volume and shape of the medial temporal lobe, accompanied by small displacements of the course of vascular structures running at its surface. A similar relationship between vessels and anatomical structure held for the hippocampus, where the subiculum, and to a lesser extent the entorhinal cortex, were affected by overlaps with the likely course of the CP or the internal carotis. Analogous considerations apply to the most posterior and medial part of BA17–18 in the occipital cortex, which may overlap with the posterior arm of the sagittal sinus (whose size varied considerably across individuals), and to the genu of the corpus callosum, especially the subgenual portion, which is located near the anterior cerebral and pericallosal arteries.

For this type of overlap, the signal from imaging modalities sensitive to vascular artifacts may in principle be improved by selecting the signal from a ROI on an individual basis, or by exploring the application of sophisticated nonlinear normalization techniques (Ashburner and Friston, 2005; Klein et al., 2009; Avants et al., 2011). In contrast, some anatomical structures were affected by the presence of vascular structures deeply embedded in brain parenchyma and hence tightly coupled with tissue. At this other extreme of the range, co-localization did not depend on misregistration of the parenchyma and the spaces containing the vascular tree. The basal ganglia, and especially the ventral striatum, were an example of this close relationship between vessels and tissue.

An intermediate position was occupied by vascular structures irrorating the cortex and running along fissures and main sulci. The large vessels branch out smaller supplying arteries in the subpial space, from which originate arterioles that penetrate the cortex (Duvernoy et al., 1981). In the cortex the presence of vascular structure was conspicuous in the medial longitudinal and the Sylvian fissures and to a less extent, in the parieto-occipital sulcus. Of particular note was the amount of vascular structures detectable anteriorly in the medial prefrontal cortex and in the inferior and posterior parts of the Sylvian fissure, affecting the insular cortex and the temporal and parietal operculum. The prominence of vascular elements in the perisylvian region observed here is consistent with the amount of signal variation attributable to vessels observed in echo-planar imaging (Lund et al., 2006) and in rest perfusion data obtained with the arterial spin labeling technique (Viviani et al., 2010). In these areas, data analysis approaches that reduce vascular confounds may be important to obtain accurate effects estimates (Glover et al., 2000; Lund et al., 2006; Chang et al., 2009). Because most sulci show large individual variability in humans (Thompson et al., 1996; Zilles et al., 1997; Kennedy et al., 1998; Van Essen and Dierker, 2007), future research may explore the effectiveness of anatomically informed (Kiebel et al., 2000) or surface-based group analysis approaches (Fischl et al., 1999; Andrade et al., 2001) that are more selective in recruiting the signal to be attributed to the cerebral cortex.

## Author Contributions

RV devised research, collected, analyzed, and wrote up the data.

## Funding

This work was supported through a NEURON-Eranet grant by the German Federal Ministry for Education and Research (BMBF, Project BrainCYP, Grant. No. 01EW1402B).

## Conflict of Interest Statement

The author declares that the research was conducted in the absence of any commercial or financial relationships that could be construed as a potential conflict of interest.

## Footnotes

^1^http://www.loni.usc.edu/Software/BrainSuite

^2^http://www.loni.usc.edu/ICBM/

^3^http://www.fil.ion.ucl.ac.uk/spm/

^4^http://www.mccauslandcenter.sc.edu/mricro/mricron/

^5^https://www.mcgill.ca/bic/software/tools-data-analysis/anatomical-mri/atlases/icbm152lin

^6^http://www.uniklinik-ulm.de/struktur/kliniken/psychiatrie-und-psychotherapie/klinik-fuer-psychiatrie-und-psychotherapie-iii-ulm/home/forschung/clinical-neuroimaging/digital-atlas.html

## References

[B1] Alsop D. C. Detre J. A. (1996). Reduced transit-time sensitivity in noninvasive magnetic resonance imaging of human cerebral blood flow. J. Cereb. Blood Flow Metab. 16, 1236–1249. 10.1097/00004647-199611000-00019 8898697

[B2] Amunts K. Kedo O. Kindler M. Pieperhoff P. Mohlberg H. Shah N. J.. (2005). Cytoarchitectonic mapping of the human amygdala, hippocampal region and entorhinal cortex: intersubject variability and probability maps. Anat. Embryol. (Berl) 210, 343–352. 10.1007/s00429-005-0025-5 16208455

[B3] Amunts K. Schleicher A. Bürgel U. Mohlberg H. Uylings H. B. M. Zilles K. (1999). Broca’s region revisited: cytoarchitecture and intersubject variability. J. Comp. Neurol. 412, 319–341. 10.1002/(sici)1096-9861(19990920)412:2<319::aid-cne10>3.0.co;2-7 10441759

[B4] Andrade A. Kherif F. Mangin J.-F. Paradis A.-L. Worsley K. J. Simon O.. (2001). Detection of fMRI activation using cortical surface mapping. Hum. Br. Mapping 12, 79–93. 10.1002/1097-0193(200102)12:2<79::aid-hbm1005>3.0.co;2-i 11169872 PMC6872103

[B5] Ashburner J. Friston K. J. (1999). Nonlinear spatial normalization using basis functions. Hum. Brain Mapp. 7, 254–266. 10.1002/(sici)1097-0193(1999)7:4<254::aid-hbm4>3.3.co;2-7 10408769 PMC6873340

[B6] Ashburner J. Friston K. J. (2005). Unified segmentation. Neuroimage 26, 839–851. 10.1016/j.neuroimage.2005.02.018 15955494

[B7] Avants B. B. Tustison N. J. Song G. Cook P. A. Klein A. Gee J. C. (2011). A reproducible evaluation of ANTs similarity metric performance in brain image registration. Neuroimage 54, 2033–2044. 10.1016/j.neuroimage.2010.09.025 20851191 PMC3065962

[B8] Biswal B. B. Mennes M. Zuo X. N. Gohel S. Kelly C. Smith S. M.. (2010). Toward discovery science of human brain function. Proc. Natl Acad. Sci. U S A 107, 4734–4739. 10.1073/pnas.0911855107 20176931 PMC2842060

[B9] Chang C. Cunningham J. P. Glover G. H. (2009). Influence of heart rate on the BOLD signal: the cardiac response function. Neuroimage 44, 857–869. 10.1016/j.neuroimage.2008.09.029 18951982 PMC2677820

[B10] Cordes D. Haughton V. M. Arfanakis K. Carew J. D. Turski P. A. Moritz C. H.. (2001). Frequencies contributing to functional connectivity in the cerebral cortex in “resting state” data. AJNR Am. J. Neuroradiol. 22, 1326–1333. 11498421 PMC7975218

[B11] Critchley M. (1930). The anterior cerebral artery and its syndromes. Brain 53, 120–165. 10.1093/brain/53.2.120 PMC2181836 19987442

[B12] Dagli M. S. Ingehold J. E. Haxby J. V. (1999). Localization of cardiac-induced signal change in fMRI. Neuroimage 9, 407–415. 10.1006/nimg.1998.0424 10191169

[B13] Dogdas B. Shattuck D. W. Leahy R. M. (2005). Segmentation of skull and scalp in 3D human MRI using mathematical morphology. Hum. Brain Mapp. 26, 273–285. 10.1002/hbm.20159 15966000 PMC6871678

[B14] Duvernoy H. M. (2005). The Human Hippocampus. Functional Anatomy, Vascularization and Serial Sections with MRI. Berlin: Springer.

[B15] Duvernoy H. M. Delon S. Vannson J. L. (1981). Cortical blood vessels of the human brain. Brain Res. Bull. 7, 519–579. 10.1016/0361-9230(81)90007-1 7317796

[B16] Eickhoff S. B. Schleicher A. Zilles K. Amunts K. (2006). The human parietal operculum I: cytoarchitectonic mapping of subdivisions. Cereb. Cortex 16, 245–267. 10.1093/cercor/bhi105 15888607

[B17] Evans A. C. Marret S. Neelin P. Collins L. Worsley K. J. Dai W.. (1992). Anatomical mapping of functional activation in stereotactic coordinate space. Neurimage 1, 43–53. 10.1016/1053-8119(92)90006-9 9343556

[B18] Feekes J. A. Cassel M. D. (2006). The vascular supply of the functional compartments of the human striatum. Brain 129, 2189–2201. 10.1093/brain/awl158 16815876

[B19] Fischl B. Sereno M. I. Tootell R. B. H. Dale A. M. (1999). High-resolution intersubject averaging and a coordinate system for the cortical surface. Hum. Brain Mapp. 8, 272–284. 10.1002/(sici)1097-0193(1999)8:4<272::aid-hbm10<3.0.co;2-4 10619420 PMC6873338

[B20] Fonov V. S. Evans A. C. Botteron K. Almli C. R. McKinstry R. C. Collins D. L.. (2011). Unbiased average age-appropriate atlases for pediatric studies. Neuroimage 54, 313–327. 10.1016/j.neuroimage.2010.07.033 20656036 PMC2962759

[B21] Friston K. J. Ashburner J. Poline J. B. Frith C. D. Heather J. D. Frackowiak R. S. J. (1996). Spatial realignment and normalization of images. Hum. Brain Mapp. 2, 165–189.

[B23] Glover G. H. Li T. Q. Ress D. (2000). Image-based method for retrospective correction of physiological motion effects in fMRI: RETROCOIR. Magn. Reson. Med. 44, 162–167. 10.1002/1522-2594(200007)44:1<162::aid-mrm23>3.3.co;2-5 10893535

[B24] Kaplan H. A. Ford D. H. (1966). The Brain Vascular System. Amsterdam: Elsevier.

[B25] Kennedy D. N. Lange N. Makris N. Bates J. Meyer J. Caviness V. S. Jr. (1998). Gyri of the human neocortex: an MRI-based analysis of volume and variance. Cereb. Cortex 8, 372–384. 10.1093/cercor/8.4.372 9651132

[B26] Kiebel S. Goebel R. Friston K. (2000). Anatomically informed basis functions. Neuroimage 11, 656–667. 10.1006/nimg.1999.0542 10860794

[B27] Kirchheiner J. Seeringer A. Viviani R. (2010). Pharmacogenetics in psychiatry. A useful clinical tool or wishful thinking for the future? Curr. Pharm. Des. 16, 136–144. 10.2174/138161210790112728 20205659

[B28] Klein A. Andersson J. Ardekani B. A. Ashburner J. Avants B. Chiang M. C.. (2009). Evaluation of 14 nonlinear deformation algorithms applied to human brain MRI registration. Neuroimage 46, 786–802. 10.1016/j.neuroimage.2008.12.037 19195496 PMC2747506

[B29] Kurth F. Eickhoff S. B. Schleicher A. Hoemke L. Zilles K. Amunts K. (2010). Cytoarchitecture and probabilistic maps of the human posterior insular cortex. Cereb. Cortex 20, 1448–1461. 10.1093/cercor/bhp208 19822572 PMC2871375

[B30] Lai S. Hopkins A. L. Haacke E. M. Li D. Wasserman B. A. Buckley P.. (1993). Identification of vascular structures as a major source of signal contrast in high resolution 2D and 3D functional activation imaging of the motor cortex at 1.5T: preliminary results. Magn. Reson. Med. 30, 387–392. 10.1002/mrm.1910300318 8412613

[B31] Lancaster J. L. Woldorff M. G. Parsons L. M. Liotti M. Freitas C. S. Rainey L.. (2000). Automated talairach atlas labels for functional brain mapping. Hum. Brain Mapp. 10, 120–131. 10.1002/1097-0193(200007)10:3<120::aid-hbm30>3.0.co;2-8 10912591 PMC6871915

[B32] Lang J. (2001). Skull Base and Related Structures: Atlas of Clinical Anatomy. Stuttgart: Schattauer.

[B33] Lund T. E. Madsen K. H. Sidaros K. Luo W.-L. Nichols T. E. (2006). Non-white noise in fMRI: does modelling have an impact? Neuroimage 29, 54–66. 10.1016/j.neuroimage.2005.07.005 16099175

[B34] Margulies D. S. Böttger J. Long X. Lv Y. Kelly C. Schäfer A.. (2010). Resting developments: a review of fmri post-processing methodologies for spontaneous brain activity. MAGMA 23, 289–307. 10.1007/s10334-010-0228-5 20972883

[B35] Margulies D. S. Kelly A. M. C. Uddin L. Q. Biswal B. B. Castellanos F. X. Milham M. P. (2007). Mapping the functional connectivity of anterior cingulate cortex. Neuroimage 37, 579–588. 10.1016/j.neuroimage.2007.05.019 17604651

[B36] Mayberg H. S. Brannan S. K. Mahurin R. K. Jerabek P. A. Brickman J. S. Tekell J. L.. (1997). Cingulate function in depression: a potential predictor of treatment response. Neuroreport 8, 1057–1061. 10.1097/00001756-199703030-00048 9141092

[B37] Mazziotta J. Toga A. Evans A. Fox P. Lancaster J. Zilles K.. (2001). A probabilistic atlas and reference system for the human brain: International Consortium for Brain Mapping ICBM. Phil. Trans. R. Soc. B Biol. Sci. 356, 1293–1322. 10.1098/rstb.2001.0915 11545704 PMC1088516

[B38] Menon R. S. Ogawa S. Tank D. W. Ugurbil K. (1993). 4 tesla gradient recalled echo characteristics of photic stimulation-induced signal changes in the human primary visual cortex. Magn. Reson. Med. 30, 380–386. 10.1002/mrm.1910300317 8412612

[B39] Morosan P. Rademacher J. Schleicher A. Amunts K. Schormann T. Zilles K. (2001). Human primary auditory cortex: cytoarchitectonic subdivisions and mapping into a spatial reference system. Neuroimage 13, 684–701. 10.1006/nimg.2000.0715 11305897

[B40] Nishimura D. G. (1990). Time-of-flight MR angiography. Magn. Reson. Med. 14, 194–201. 10.1002/mrm.1910140206 2345502

[B41] Perona P. Malik J. (1990). Scale-space and edge detection using anisotropic diffusion. IEEE Trans. Patt. Anal. Mach. Intell. 12, 629–639. 10.1109/34.56205

[B42] Poldrack R. A. (2007). Regions of interest analysis for fMRI. Soc. Cogn. Affect. Neurosci. 2, 67–70. 10.1093/scan/nsm006 18985121 PMC2555436

[B43] Shattuck D. W. Leahy R. M. (2002). BrainSuite: an automated cortical surface identification tool. Med. Image Anal. 8, 129–142. 10.1016/s1361-8415(02)00054-3 12045000

[B44] Simon J. H. Li D. Traboulsee A. Coyle P. K. Arnold D. L. Barkhof D. L.. (2006). Standardized MR imaging protocol for multiple sclerosis: consortium of MS centers consensus guidelines. AJNR Am. J. Neuroradiol. 27, 455–461. 16484429 PMC8148806

[B45] Stefani M. A. Schneider F. L. Marrone A. C. H. Severino A. G. Jackowski A. P. Wallance M. C. (2000). Anatomic variations of anterior cerebral artery cortical branches. Clin. Anat. 13, 231–236. 10.1002/1098-2353(2000)13:4<231::aid-ca1>3.0.co;2-t 10873213

[B46] Thompson P. M. Schwartz C. Lin R. T. Khan A. A. Toga A. W. (1996). Three-dimensional statistical analysis of sulcal varibility in the human brain. J. Neurosci. 16, 4261–4274. 8753887 10.1523/JNEUROSCI.16-13-04261.1996 PMC6578992

[B47] Toga A. W. Thompson P. M. Mori S. Amunts K. Zilles K. (2006). Towards multimodal atlases of the human brain. Nat. Rev. Neurosci. 7, 952–966. 10.1038/nrn2012 17115077 PMC3113553

[B48] Türe U. Yaşargil G. Al-Mefty O. Yaşargil D. C. H. (2000). Arteries of the insula. J. Neurosurg. 92, 676–687. 10.3171/jns.2000.92.4.0676 10761659

[B49] Ugur H. C. Kahilogullari G. Esmer A. F. Comert A. Odabasi A. B. Tekdemir I.. (2006). A neurosurgical view of anatomical variations of the distal anterior cerebral artey: an anatomical study. J. Neurosurg. 104, 278–284. 10.3171/jns.2006.104.2.278 16509502

[B50] Van Dijk K. R. A. Hedden T. Venkataraman A. Evans K. C. Lazar S. W. Buckner R. L. (2010). Intrinsic functional connectivity as a tool for human connectomics: theory, properties and optimization. J. Neurophysiol. 103, 297–321. 10.1152/jn.00783.2009 19889849 PMC2807224

[B51] Van Essen D. C. Dierker D. L. (2007). Surface-based and probabilistic atlases of primate cerebral cortex. Neuron 56, 209–225. 10.1016/j.neuron.2007.10.015 17964241

[B52] Viviani R. Beschoner P. Ehrhard K. Schmitz B. Thöne J. (2007). Non-normality and transformations of random fields, with an application to voxel-based morphometry. Neuroimage 35, 121–130. 10.1016/j.neuroimage.2006.11.037 17222566

[B53] Viviani R. Beschoner P. Lo H. Osterfeld N. Thöne J. Sim E. J. (2010). Components of acquisition-to-acquisition variance in continuous arterial spin labelling (CASL) imaging. BMC Neurosci. 11:30. 10.1186/1471-2202-11-30 20196843 PMC2841194

[B54] Viviani R. Sim E. J. Lo H. Richter S. Haffer S. Osterfeld N.. (2009). Components of variance in brain perfusion and the design of studies of individual differences: the baseline study. NeuroImage 46, 12–22. 10.1016/j.neuroimage.2009.01.041 19457381

[B55] Vogt B. A. Nimchinsky E. A. Vogt L. J. Hof P. R. (1995). Human cingulate cortex: surface features, flat maps and cytoarchitecture. J. Comp. Neurol. 359, 490–506. 10.1002/cne.903590310 7499543

[B56] Wehrli F. W. (1990). Time-of-flight effects in MR imaging of flow. Magn. Reson. Med. 14, 187–193. 10.1002/mrm.1910140205 2345501

[B57] Zilles K. Schleicher A. Langemann C. Amunts K. Morosan P. Palomero-Gallagher N.. (1997). Quantitative analysis of sulci in the human cerebral cortex: development, regional heterogeneity, gender difference, asymmetry, intersubject variability and cortical architecture. Hum. Brain Mapp. 5, 218–221. 10.1002/(sici)1097-0193(1997)5:4<218::AID-HBM2>3.3.CO;2-h 20408218

